# Ataxia telangiectasia mutated: The potential negative regulator in platelet-derived growth factor-BB promoted proliferation of pulmonary arterial smooth muscle cells

**DOI:** 10.3389/fcvm.2022.942251

**Published:** 2022-08-03

**Authors:** Chaoyi Qin, Yiheng Zan, Liang Xie, Hanmin Liu

**Affiliations:** ^1^Department of Cardiovascular Surgery, West China Hospital, Sichuan University, Chengdu, China; ^2^Pulmonary Vascular Remodeling Research Unit, West China Institute of Women's and Children's Health, West China Second University Hospital, Sichuan University, Chengdu, China; ^3^Key Laboratory of Obstetric and Gynecologic and Pediatric Disease, Chengdu, China; ^4^Department of Pediatrics, West China Second University Hospital, Sichuan University, Chengdu, China; ^5^Department of Burns and Plastic Surgery, West China Hospital, Sichuan University, Chengdu, China

**Keywords:** ataxia telangiectasia mutated, pulmonary arterial hypertension, smooth muscle cell, proliferation, ROS

## Abstract

**Objective:**

To study the role of ataxia telangiectasia mutated (ATM) in the platelet-derived growth factor (PDGF)-BB-induced proliferation of pulmonary arterial smooth muscle cells (PASMCs) through reactive oxygen species (ROS) formation.

**Methods:**

Primary cultures of PASMCs were treated with different concentrations of PDGF-BB or exogenous hydrogen peroxide (H_2_O_2_). The activation level of ATM and the proliferation level of PASMCs were measured by immunofluorescence staining and Cell Counting Kit-8, respectively. Moreover, NADPH oxidase 2 (NOX2) and intracellular H_2_O_2_ were detected under the stimulation of different levels of PDGF-BB by Western blot and dihydroethidium staining.

**Results:**

Both the control group and 50 ng/ml of the PDGF-BB group showed significantly higher levels of phosphorylation ATM compared to other groups (*P* < 0.05). With the ATM inhibitor, 50 ng/ml of the PDGF-BB group showed further increased proliferative level compared to the 10 ng/ml (*P* < 0.05). Both the levels of NOX2 and H_2_O_2_ showed dose-dependent manners under PDGF-BB stimulation (*P* < 0.05). ATM could be activated by H_2_O_2_ upon a dose-dependent way, except for the 500 μM H_2_O_2_ group. Under 200 μM H_2_O_2_ stimulation, proliferation level decreased significantly (*P* < 0.05), while no significant difference was shown with the addition of ATM inhibitor (*P* > 0.05).

**Conclusion:**

Our study first established ROS-induced ATM activation in PDGF-BB-stimulated proliferation of PASMCs. Inhibition of ATM had promoted effects on the proliferation of PASMCs under the excessive levels of PDGF-BB and H_2_O_2_. Our study might provide a novel promising target for the treatment of pulmonary arterial hypertension (PAH).

## Introduction

Pulmonary arterial hypertension (PAH) is a chronic disease, characterized by the narrowing and obstruction of the small pulmonary arteries, which results in increased pulmonary vascular resistance, right heart failure, and probably causes death (1). Although many studies are conducted to illustrate the pathogenesis of PAH, to fully understand that still needs a long way to approach. One of the major pathogenic mechanisms is pulmonary vascular remodeling, featured pathologically by the enhanced proliferation of endothelial cells and pulmonary artery smooth muscle cells (PASMCs) (2, 3).

Ataxia telangiectasia mutated (ATM) is a serine/threonine kinase. Together with DNA-dependent protein kinase catalytic subunits (DNA-PKcs) and ATM- and Rad3-related (ATR), these three are members of the phosphatidylinositol-3 kinase-like protein kinase (PIKK) family. When DNA damages occur, these proteins can activate the repair mechanism and suspend the duplication by activating the cell cycle checkpoint (4, 5). ATM is fully studied as an essential regulator for the proliferation, migration, and invasion of cancer cells (6, 7). In addition, it is reported that ATM could be activated directly by reactive oxygen species (ROS) regardless of the DNA damage (8). Oxidative stress is associated with a broad range of pathological states, including carcinogenesis, ischemia-reperfusion injury, and pulmonary vascular remodeling (9, 10). ROS is widely generated in all the healthy cells in pulmonary vasculatures and is involved in regulating many physiological cellular processes (11). Excessive ROS generated by activated NADPH oxidase 2 (NOX2) and mitochondria results in activating many intracellular signaling pathways, partially contributing to PAH formation (12). Importantly, ROS is further studied as a therapeutic target regarding for the treatment of PAH (13).

Platelet-derived growth factor (PDGF) can act with PDGF receptor to promote the proliferation of human PASMCs. A clinical trial evaluating the PDGF receptor inhibitor has been carried out for treating PAH (14). In the PDGF-induced proliferation and migration of vascular smooth muscle cells, accumulation of ROS is also observed (15), suggesting that ROS may play a role in PDGF-induced proliferation of PASMCs. However, acting as a sensor for ROS, how ATM exerts its role in regulating the proliferation of PASMCs has not been investigated.

Thus, we conduct a PDGF-BB-induced PASMC proliferation system to study the role of ATM through the addition of ATM inhibitor, along with detecting the NADPH/ROS signaling activation under different concentrations of PDGF-BB stimulation. Furthermore, we use different levels of hydrogen peroxide (H_2_O_2_) to stimulate the PASMCs under ATM inhibition to figure out the role of ATM in PASMCs proliferation.

## Methods

### Isolation and culture of pulmonary artery smooth muscle cells

**Figure 1:**
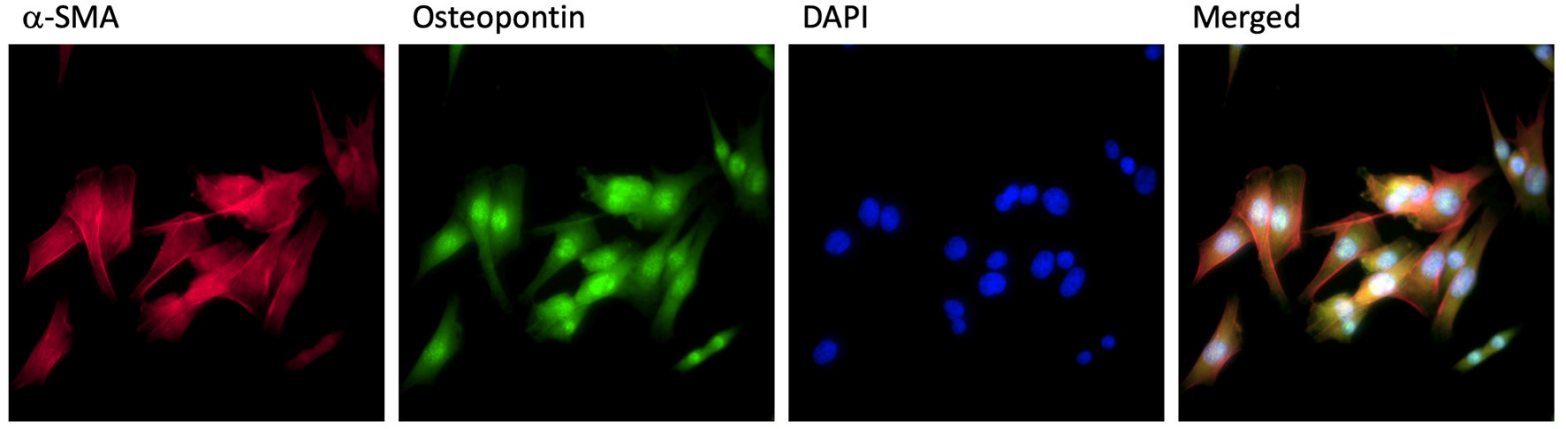
PASMCs identification. PASMCs were stained by α-SMA antibody (red), osteopontin antibody (green), DAPI (blue), and merged in the same image.

All the procedures followed our previous study (16). Briefly, male Sprague–Dawley (SD) rats obtained from the Animal Breeding Center of Sichuan University were sacrificed by the cervical dislocation. The proximal part (~0.5 cm) of the pulmonary artery was collected carefully under aseptic conditions. The adventitia of the pulmonary artery with connective and fat tissues was removed and the media of the pulmonary artery was cut into small pieces in the phosphate-buffered saline (PBS). These small pieces were cultured in Dulbecco's Modified Eagle Medium (DMEM) with 10% fetal bovine serum (FBS), 100 U/ml penicillin, and 100 mg/ml streptomycin at 37°C in a humidified 5% CO_2_ atmosphere. Primary PASMCs were subcultured to the third generation and readied for the experiments. Anti-α-smooth muscle antibody (SMA) and antiosteopontin (OPN) antibodies were used to confirm the PASMCs (Figure 1). This study was conducted and registered by the Ethical Review Board at Sichuan University.

### Experimental protocols of platelet-derived growth factor-BB and hydrogen peroxide treatments

**Figure 2:**
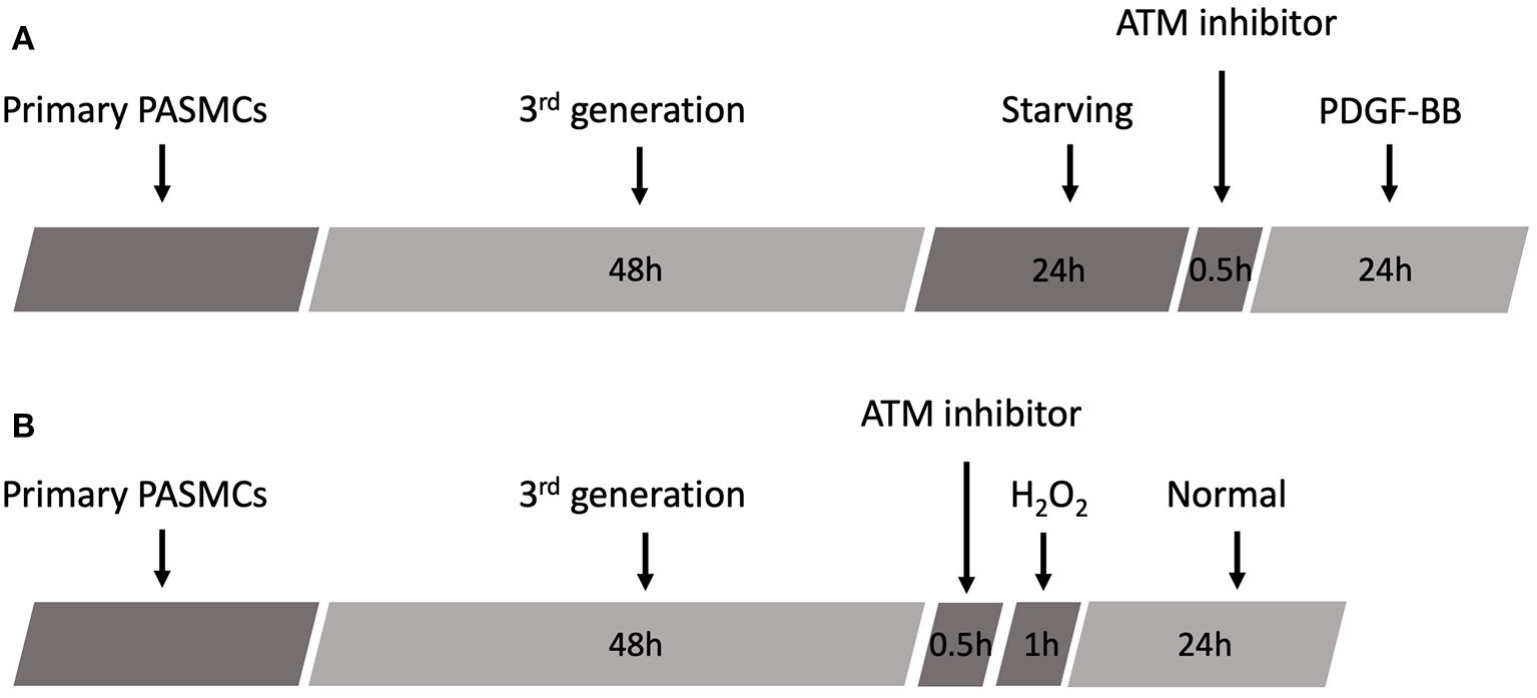
Schematic of experimental design. **(A)** Schematic protocol of PDGF-BB treatment. **(B)** Schematic protocol of H_2_O_2_ treatment.

Platelet-derived growth factor-BB was obtained from the R&D Systems (R&D Systems Incorporation, USA). The third generation of PASMCs was treated with PDGF-BB for 24 h, following the starvation for 24 h in DMEM without FBS. The concentrations of PDGF-BB were 2, 10, and 50 ng/ml, while the same volume of PBS was added as the control group. A total of 3 μM of ATM inhibitor was administrated for 30 min prior to PDGF-BB treatment. After 24 h treatment, PASMCs were harvested for the next experiments (Figure 2A).

Hydrogen peroxide was supplied from Sigma (Sigma Corporation, USA). H_2_O_2_ was prepared into 20, 100, 200, and 500 μM with serum-free DMEM. The third generation of PASMCs was cultured in different concentrations of H_2_O_2_ for 1 h with or without ATM inhibitor pretreatment, followed by the replacement of the medium to normal DMEM with 10% FBS for another 24 h. Then, PASMCs were harvested for the next experiments (Figure 2B).

### *In-situ* detection of extracellular hydrogen peroxide

${\text{O}}_{2}^{-}$ generation was determined by dihydroethidium (DHE) (Invitrogen) staining (17). The third generation of PASMCs was cultured on the cover glass for a 48-well plate. Diphenyleneiodonium (DPI) was used as the inhibitor for NOX2. PASMCs were incubated with DPI (30 μM) or PBS at 37°C for 30 min, followed by culturing in DHE (2 μM) at 37°C for 30 min in a dark, humidified chamber. After washing with PBS, red fluorescence was captured at 400X magnification by a fluorescent microscope with a digital camera. The fluorescent intensities were measured by AxioVision software.

### Proliferation measurement by cell counting Kit-8 assay

Cell Counting Kit-8 (CCK-8) was purchased from the Dojindo (Dojindo Molecular Technology Incorporation, USA). According to the manufacturer's instruction, the third generation of PASMCs was cultured in a 96-well plate. After specific treatments, 10 μl of the CCK-8 solution was added to each well, followed by 2 h incubation at 37°C with a humidified 5% CO_2_. Absorbance was measured by a microplate reader under 450 nm.

### Detection of phosphorylation of ataxia telangiectasia mutated

Immunofluorescence Staining Kit was purchased from Beyotime (Beyotime, China). The third generation of PASMCs was cultured on the cover glass for a 48-well plate. All the procedures are strictly followed by the manufacturer's instructions. Briefly, cells are fixed and blocked by using the fixing buffer and blocking buffer, respectively. Antiphosphorylated ATM antibody (ab36810, Abcam, USA) was used for incubation and detection at 4°C overnight. After removal of the primary antibody, a specific fluorescent enzyme-connected secondary antibody was used for 60 min in a dark chamber. The fluorescent microscope was used to detect the phosphorylated ATM, which submitted the red fluorescence.

### Western blot analysis for NADPH oxidase 2 protein measurement

Pulmonary artery smooth muscle cells were washed three times with PBS on the ice and lysed by NP40 lysis buffer [50 mM Tris-HCl (pH = 7.4), 1% Nonidet P-40, and 150 mM NaCl] for 30 min. Cell lysates were collected and centrifuged at 12,000 g for 5 min at 4°C. The supernatant was kept and determined by BCA Protein Assay Kit (Beyotime, China). Proteins (50 μg/lane) were added and separated by electrophoresis on 10% polyacrylamide sodium dodecyl sulfate gels, followed by transfer to polyvinylidene fluoride (PVDF) membranes. The membranes were incubated with anti-NOX2 antibody at 4°C overnight and a secondary antibody for 60 min at room temperature. The blots were developed using the enhanced chemiluminescence (ECL) Western Blotting Detection System with a ChemiDoc^TM^ MP Imager (Bio-Rad, USA). The densitometry values were measured using the FluorChem 8,000 software (Alpha Innotech, California, USA).

### Statistical analysis

All the data were presented as mean ± SEM. One-way ANOVA followed by the Bonferroni test was used to examine the statistically significant differences between multiple groups. *P* < 0.05 was considered significantly different.

## Results

### Phosphorylation of ataxia telangiectasia mutated inhibited proliferation of pulmonary artery smooth muscle cells under high concentration of platelet-derived growth factor-BB stimulation

**Figure 3:**
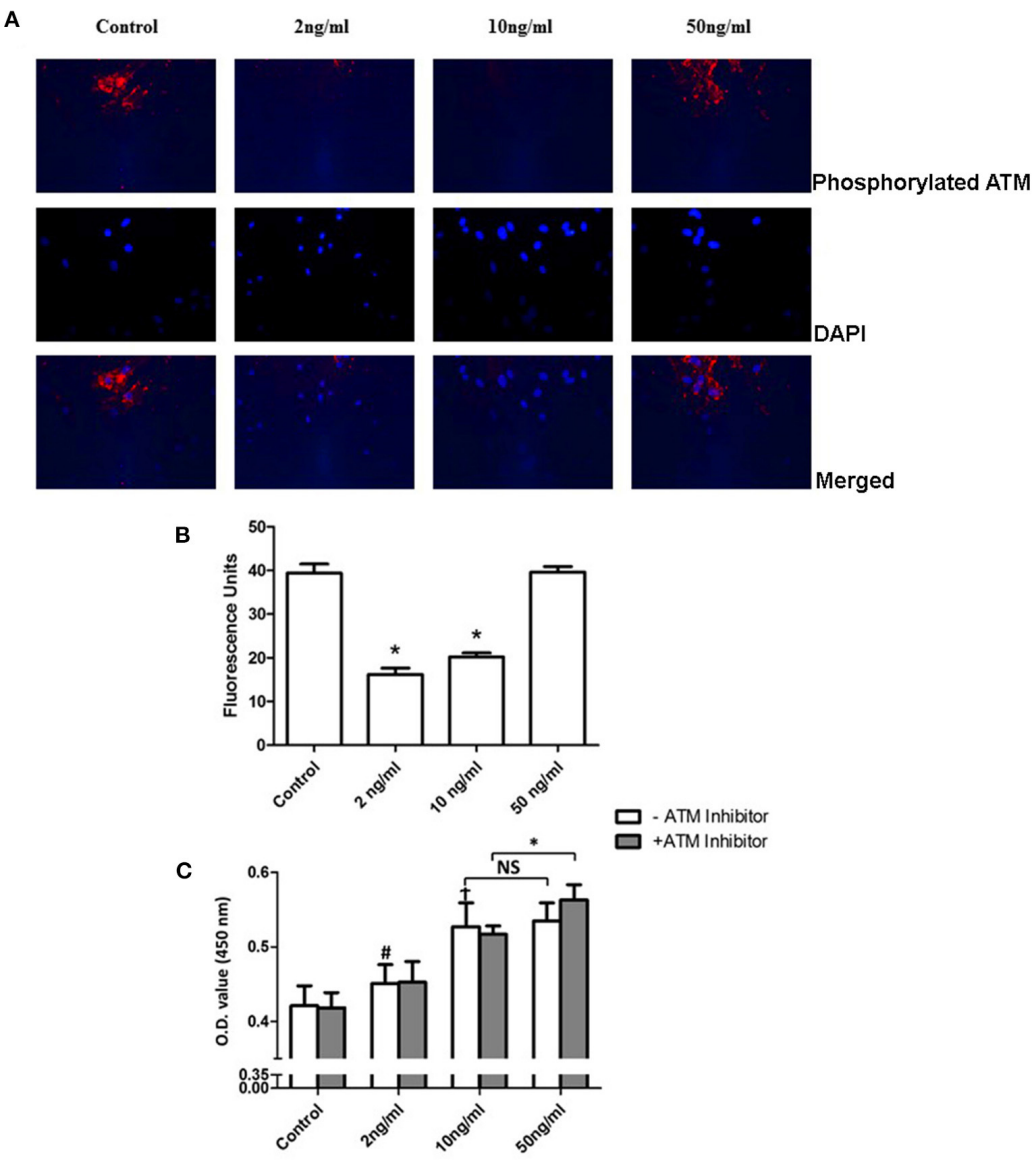
Phosphorylation of ATM inhibited proliferation of PASMCs under a high concentration of PDGF-BB stimulation. **(A)** Phosphorylation of ATM levels of PASMCs under different concentrations. PDGF-BB was shown in the first panel. Images of DAPI staining for the nucleus were shown in the middle panel. The merged images were represented in the last panel. **(B)** Quantification of the phosphorylation level of PASMCs showed in the bar graph. Both the 2 and 10 ng/ml of PDGF-BB groups showed a significant lower level of phosphorylation of ATM (*P* < 0.05). **(C)** Cell Counting Kit-8 results showed that the 2, 10, and 50 ng/ml groups had significantly higher proliferation levels compared to the control group (*P* < 0.05). With the pretreatment of ATM inhibitor, a significant increase was found between the 10 and 50 ng/ml groups (*P* < 0.05). *N* = 5. *P < 0.05 vs. control. ^#^*p* < 0.05, vs. Control.

Pulmonary artery smooth muscle cells were identified by staining of α-SMA, osteopontin, and DAPI. Staining images and merged images are shown in Figure 1. To examine the phosphorylation level of ATM and proliferation level of PASMCs under the stimulation of PDGF-BB with or without ATM inhibitor, pulmonary arteries were captured from young SD rats and cultured for the third generation. Thirty min pretreatment of ATM inhibitor (KU-60019, obtained from Selleck Chemicals, USA) or PBS was performed after 24 h starving, followed by the addition of different concentrations of PDGF-BB for 24 h (Figure 2A). PASMCs were fixed and prepared for detecting the phosphorylation of ATM. As shown in Figure 3A, phosphorylated ATM, nucleus, and merged images were presented. The intensities of fluorescence of phosphorylated ATM are shown in Figure 3B. The control group and 50 ng/ml of the PDGF-BB group showed higher levels of phosphorylation of ATM, while low levels of phosphorylated ATM were presented in 2 and 10 ng/ml of the PDGF-BB groups (*P* < 0.05). Further, proliferative level of PASMCs was measured by CCK-8 assay. Without the pretreatment of ATM inhibitor, 2, 10, and 50 ng/ml of groups showed a significant increase in proliferation compared with the control group. There is no significant difference identified between 10 and 50 ng/ml of groups. With the pretreatment of ATM inhibitor, proliferative levels of PASMCs under PDGF-BB treatment were not affected. Interestingly, a slight but significant difference was found between 10 and 50 ng/ml of groups (*P* < 0.05, Figure 3C). These data suggested that a low concentration of PDGF-BB stimulation might inhibit the phosphorylation of ATM and ATM inhibitor could improve the proliferative effect of a high concentration of PDGF-BB.

### Platelet-derived growth factor-BB stimulation increased the expression of nadph oxidase 2 under a dose-dependent manner

**Figure 4:**
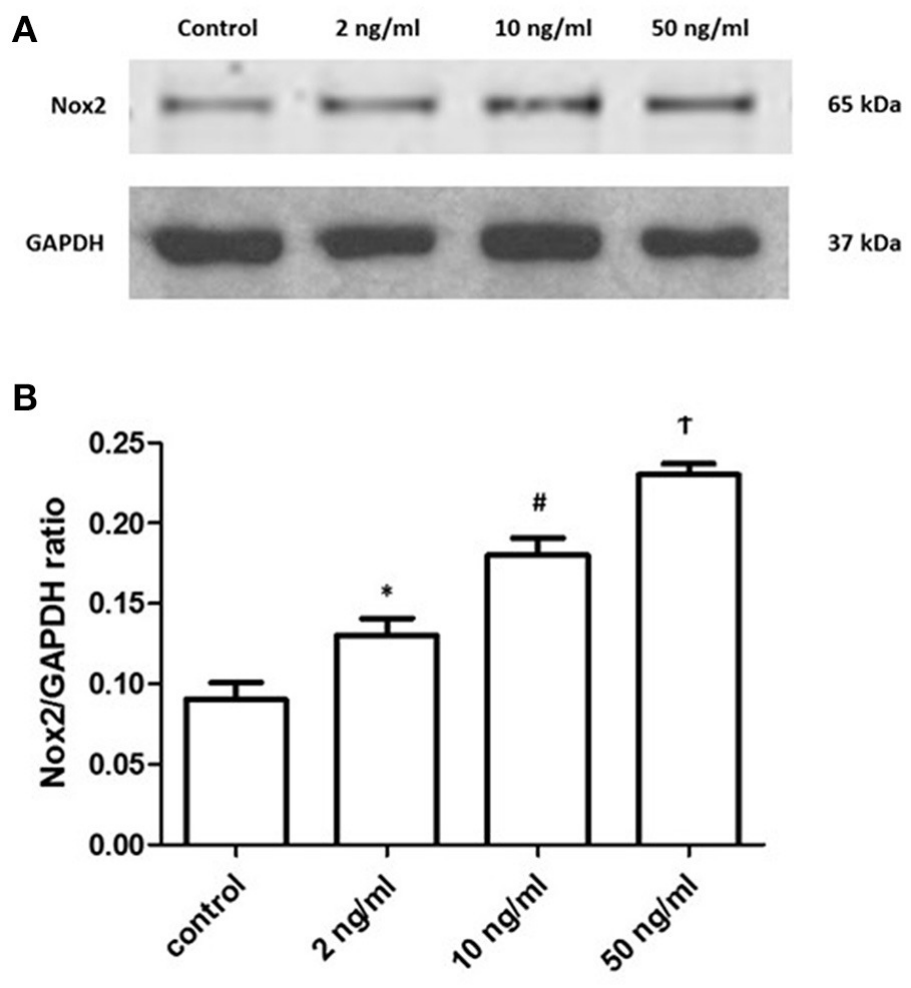
PDGF-BB stimulation increased the expression of NOX2 under a dose-dependent manner. **(A)** Representative images of the expression of NOX2 under the stimulation of PDGF-BB were shown. **(B)** The ratios of NOX2 to GAPDH were shown. The expression of NOX2 showed the dose-dependent manner under the PDGF-BB stimulation (*P* < 0.05). *N* = 4. *P < 0.05 vs. control; ^#^*P* < 0.05 vs. 2 ng/ml group; ^†^*P* < 0.05 vs. 10 ng/ml group.

Many studies revealed that NOX2 could produce H_2_O_2_ under different stimulations (12). Additionally, studies about ATM reported that ATM can be phosphorylated by H_2_O_2_ (18). Therefore, we analyze the NOX2 level under the stimulation of different PDGF-BB concentrations. After 24 h treatment of PDGF-BB, PASMCs were collected and prepared for Western blots. Representative images are shown in Figure 4A and the quantification bar chart is shown in Figure 4B. With the increase of PDGF-BB concentrations, the expression of NOX2 became significantly higher (*P* < 0.05). Our data suggested that PDGF-BB can increase the expression of NOX2 in a dose-dependent manner.

### Platelet-derived growth factor-BB stimulation increased the formation of hydrogen peroxide under a dose-dependent manner

**Figure 5:**
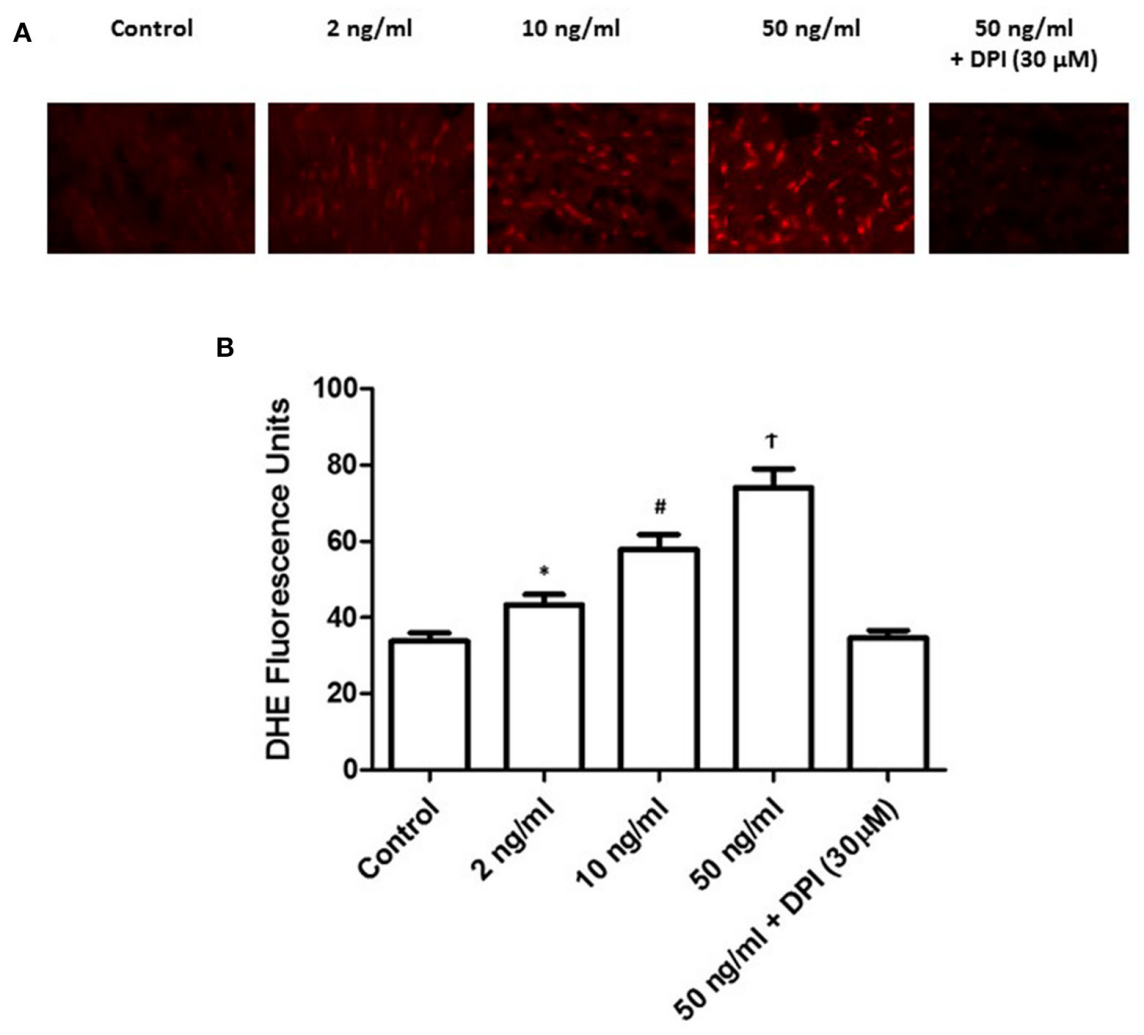
PDGF-BB stimulation increased the formation of H_2_O_2_ under a dose-dependent manner. **(A)** Representative images of DHE staining were shown. **(B)** Quantification of the fluorescence intensity was calculated. Under different concentration of PDGF-BB, H_2_O_2_ level increased significantly with the increase of PDGF-BB (*P* < 0.05). *N* = 4–6. *P < 0.05 vs. control; ^#^*P* < 0.05 vs. 2 ng/ml group; ^†^*P* < 0.05 vs. 10 ng/ml group.

To further detect the level and its resource, we treated the primary PASMCs culture with different concentrations of PDGF-BB following 24 h starving. We also set the diphenyleneiodonium (DPI) group, which contained the 30 min pretreatment of DPI followed by 50 ng/ml of PDGF-BB treatment. DHE staining was performed to detect the H_2_O_2_ level. As shown in Figure 5, with the stimulation of PDGF-BB, H_2_O_2_ level increased significantly (*P* < 0.05). More important, after the pretreatment of DPI, H_2_O_2_ level did not elevate after PDGF-BB stimulation (*P* < 0.05). These data demonstrated that PDGF-BB stimulation can promote the H_2_O_2_ accumulation and DPI, NOX2 inhibitor, can abolish this effect, which means that NOX2 took responsibility for the formation of H_2_O_2_.

### Inhibition of ataxia telangiectasia mutated promoted the proliferation of pulmonary artery smooth muscle cells under a high concentration of extracellular hydrogen peroxide

**Figure 6:**
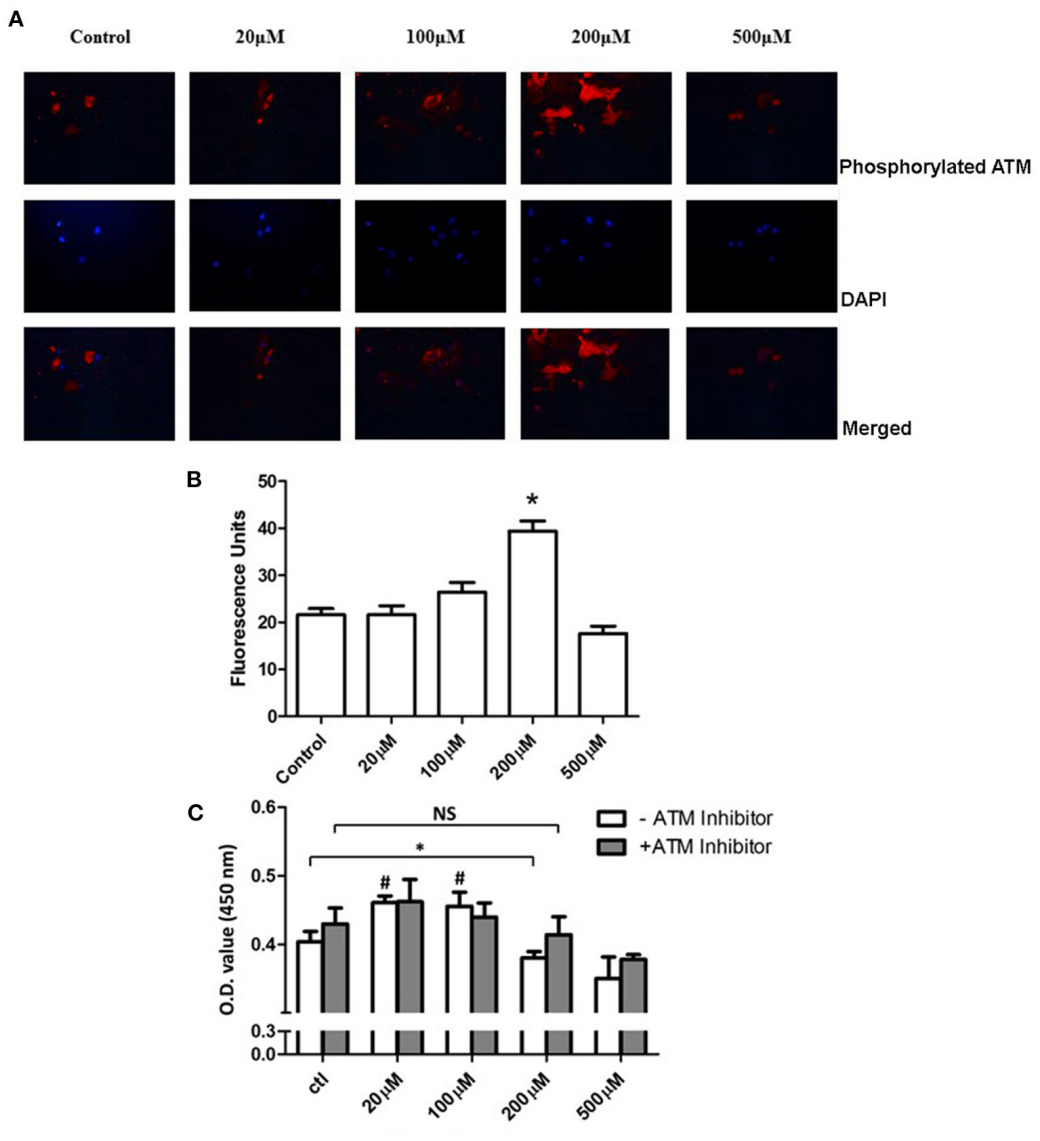
Inhibition of ATM promotes the proliferation of PASMCs under a high concentration of extracellular H_2_O_2_. **(A)** Immunohistochemistry staining of ATM under PDGF-BB stimulation was shown in the first panel. Images of DAPI staining for the nucleus were shown in the middle panel. The merged images were represented in the last panel. **(B)** Quantification data of intensities of images were shown. The intensity of the 200 μM group showed significantly higher than the control group (*P* < 0.05). **(C)** Cell Counting Kit-8 results showed that the 20 and 100 μM groups had significantly higher proliferation levels compared to the control group (*P* < 0.05). Under 200 μM of H_2_O_2_ stimulation, significantly decreased proliferation was discovered (*P* < 0.05). With the pretreatment of ATM inhibitor, no further significant difference was found between 200 μM of H_2_O_2_ and the control group (*P* > 0.05). *N* = 5. ^#^*P* < 0.05 vs. control. *P < 0.05 vs. control.

To understand the role of H_2_O_2_ on PASMCs, we incubate the primary PASMCs with extra H_2_O_2_ (Figure 2B). After 24 h starvation of the PASMCs, different concentrations of H_2_O_2_ were added into the cell culture medium with a serum-free DMEM medium. After 1 h incubation at 37°C with 5% CO_2_, the H_2_O_2_ medium was replaced by a fresh cell culture medium and cultured for another 24 h. PASMCs were collected and prepared for detecting the phosphorylation of ATM and CCK-8 assay. As shown in Figure 6A, 100 μM H_2_O_2_ stimulation could partially activate the phosphorylation of ATM and 200 μM H_2_O_2_ significantly activated the phosphorylation of ATM. Figure 6B demonstrates the intensity of fluorescence of phosphorylated ATM, which showed that an increasing intensity with the concentration of H_2_O_2_ except for the 500 μM concentration, and the 100 and 200 μM group had the significant phosphorylation of ATM (*P* < 0.05). Proliferation study (CCK-8 assay) revealed interesting results (Figure 6C). Lower levels (20 and 100 μM) of H_2_O_2_ could significantly promote the proliferation of PASMCs compared to the control group (*P* < 0.05). With the stimulation of 200 μM H_2_O_2_, proliferation level decreased significantly (*P* < 0.05). However, with the addition of ATM inhibitor, proliferation level under 200 μM H_2_O_2_ did not show a further decrease compared with the control group (*P* > 0.05).

## Discussion

Our study first brought ATM mechanism into PAH by detecting ATM activation in PDGF-BB-stimulated proliferation of PASMCs. We revealed that the optimal level of PDGF-BB could activate the limited amount of ROS formation, which inhibited the phosphorylation of ATM and promoted the proliferation of PASMCs. However, a high concentration of PDGF-BB could not promote the proliferation of PASMCs, which might be caused by the accumulation of excessive ROS formation. Intriguingly, with the ATM inhibitor, a high level of PDGF-BB showed additional positive effects on the proliferation of PASMCs.

Ataxia telangiectasia mutated is first identified in patients with ataxia telangiectasia, characterized by the extreme cellular sensitivity to radiation and a predisposition to cancer (19). Activation of ATM is responsible for the repair of damaged DNA double-strand and acts as a checkpoint to stop improper duplication (20). ATM is well-investigated as the key factor in responding to double-strand breaks, which can interact with the Mre11/Rad50/Nbs1 (MRN) complex at damage sites (21). Since the dysfunction of DNA repair mechanisms will deteriorate the accumulation the DNA errors and genomic instability, which is a well-known indicator of cancer, the disorder of ATM function has been a hot topic in the field of oncology (22).

Recently, a review article revealed that the proliferation of PASMCs in pulmonary arterial hypertension showed many cancer-like characteristics. PASMCs showed similar patterns compared with the features of cancer cells, including cellular metabolic disorder, self-sufficiency in growth signals, insensitivity to antigrowth signals, limitless replicative potential, sustained angiogenesis, and evading apoptosis (23). In the present study, we used the *in-vitro* PAH model to mimic the proliferation of PASMCs. Under different stimulation levels of PDGF-BB, we found the different activation levels of ATM. Interestingly, the ATM inhibitor could discharge the plateau effect of a high concentration of PDGF-BB and further promote the proliferation of PASMCs under the high level of PDGF-BB. In addition, under the low level of PDGF-BB stimulation, ATM kept inactivated status, while ATM was activated under serum-free conditions. Our study suggested that the functional status of ATM at least partially determines the proliferation of PASMCs with the stimulation of PDGF-BB.

Ataxia telangiectasia mutated is reported to not only respond to the DNA double-strand breaks, but also be activated under oxidative stress (24). It is reasonable that the instability of DNA may play a role in the pathogenesis of PAH, while oxidative stress is well-accepted as a contributor to PAH. Many studies demonstrate that ROS can activate multiple cellular signaling pathways to promote the proliferation of both endothelial cells and PASMCs. Therefore, ROS is reported to be a therapeutic target in PAH (13, 25). To study the mechanism of ATM in PDGF-BB-stimulated PASMCs, we used the additional extracellular H_2_O_2_. In our experiments, a lower level of H_2_O_2_ (< 200 μM) could promote the proliferation of PASMCs without activation of ATM. However, a higher level of H_2_O_2_ had negative effects on the proliferation of PASMCs, which could also activate the ATM. Surprisingly, with the inhibitor of ATM, 200 μM of H_2_O_2_ had no longer negative effects on the proliferation of PASMCs.

Many studies demonstrated that dysfunction of mitochondria, activation of NOX family, and uncoupled NOS contribute to the ROS formation in PAH (26). It is studied that PDGF-BB couldinduce intracellular ROS generation, which can be attenuated by the inhibitors of the NOX family (27). It is also believed that NOX2 is the most widely presented vascular NOX isoform, which can be detected in PASMCs, adventitial fibroblasts, endothelial cells, and perivascular adipocytes (12). In our study, we further detected the activation level of NOX2 and the intracellular H_2_O_2_ level under different concentrations of PDGF-BB stimulation. Both the activation level of NOX2 and H_2_O_2_ level showed a dose-dependent way of responding to the stimulation of PDGF-BB. Taken together, we suggested that PDGF-BB could induce ROS through NOX2 activation. With the accumulation of excessive ROS, ATM can be activated to call for the cell cycle arrest to stop the proliferation of PASMCs. More importantly, with the ATM inhibitor, a higher proliferation level of PASMCs was shown compared to the same condition without the ATM inhibitor.

## Conclusion and future perspective

To summarize our study, we first established the role of ATM in the PDGF-BB-stimulated proliferation of PASMCs. The generation of ROS from NOX2 was involved in this new pathological mechanism in PAH. Our study might provide a new pharmacological target for the treatment of PAH, which was to restore the ATM function to prevent the pathological proliferation of PASMCs. According to the study in oncology, we hypothesized that the genetic dysfunction of ATM might take responsibility for PAH, which needs further clinical data about patients with PAH to confirm.

## Data availability statement

The raw data supporting the conclusions of this article will be made available by the authors, without undue reservation.

## Ethics statement

The animal study was reviewed and approved by the Ethics Review Boards at the Sichuan University.

## Author contributions

CQ and YZ accomplished most cell studies and biological studies. CQ wrote this manuscript. LX helped cell studies and biological studies. HL designed the study and supervised the whole experiments. All authors contributed to the article and approved the submitted version.

## Funding

This study was supported by the National Scientific Foundation of China (81700311).

## Conflict of interest

The authors declare that the research was conducted in the absence of any commercial or financial relationships that could be construed as a potential conflict of interest.

## Publisher's note

All claims expressed in this article are solely those of the authors and do not necessarily represent those of their affiliated organizations, or those of the publisher, the editors and the reviewers. Any product that may be evaluated in this article, or claim that may be made by its manufacturer, is not guaranteed or endorsed by the publisher.
